# Mental wellbeing in crisis line volunteers: understanding emotional impact of the work, challenges and resources. A qualitative study

**DOI:** 10.1080/17482631.2021.1986920

**Published:** 2021-10-25

**Authors:** Renate Catharina Wilhelmina Johanna Willems, Constance Hélene Christine Drossaert, Patricia Vuijk, Ernst Thomas Bohlmeijer

**Affiliations:** a Rotterdam University of Applied Sciences, Research Centre Innovations in Care, Rotterdam, the Netherlands; b University of Twente, Department of Psychology, Health and Technology, Enschede, the Netherlands

**Keywords:** Crisis line volunteers, emotional impact, challenges, resources, qualitative research

## Abstract

**Background:**

: Crisis line volunteers are a valuable addition to formal care. Although there is growing evidence of decreased mental wellbeing of crisis line volunteers, a comprehensive overview of perceived emotional impact from the perspective of volunteers is lacking. Purpose: This study explores the emotional impact, the challenges that crisis line volunteers encounter, and the resources that they use to cope with these challenges.

**Method:**

: A grounded theory approach was used to explore the subjective experiences of the participants. Four focus groups with twentytwo active volunteers and eight interviews with former volunteers were conducted.

**Results:**

: Results provide an overview of emotions that volunteers experience in their work. (e.g., gratification, compassion, frustration, and powerlessness). Challenges are related to the characteristics of callers (e.g., inappropriate behaviour) and topics of the calls (e.g., suicidality). Resources to cope adequately with negative emotions are, among others, a self-compassionate attitude and good training.

**Conclusion:**

: This study highlights the importance of training of volunteers in dealing with specific callers, and gives input for the development of interventions aimed at increasing personal resources, such as awareness of positive emotions and self-compassion. These resources can help to increase the mental wellbeing of crisis line volunteers and reduce turn-over rates.

## Introduction

Crisis line services offer anonymous emotional support by telephone or chat to anyone who cannot or does not want to use formal care. In Europe, more than 21.000 crisis line volunteers, who are trained to be non-judgemental, empathetic, respectful, and caring, conduct over five million telephone calls and 130.000 chat and email conversations each year (Székely et al., 2015). Most crisis line services use the philosophy of non-intervention: a listening ear is offered, but no therapeutic intervention is applied (IFOTES, 2020; Székely et al., 2015). Research has provided some evidence on the effectiveness of crisis line services. For example, callers reported lower levels of distress during and after the call (Gould et al., 2007; Kalafat et al., 2007) and crisis line services have even been shown to be effective in preventing suicidality (Gould et al., 2007; King et al., 2003). Therefore, crisis line services are an important addition to the existing care.

Although the work at a crisis line service is highly satisfactory to crisis line volunteers (Aguirre & Bolton, 2013; Hellman & House, 2006), volunteers are confronted with a high variety of complex topics, such as suicidality, loneliness, and abuse (Gould et al., 2007; Kalafat et al., 2007; Pollock et al., 2013). In addition, some callers show inappropriate behaviours such as calling the volunteer names, calling multiple times without clear reason, or calling for sexual gratification purposes (Pirkis et al., 2016; Pollock et al., 2013). Continuously switching between complex topics and simultaneously coping with inappropriate behaviour of callers require a great mental flexibility from volunteers and may eventually have a negative impact on their mental wellbeing.

Paying attention to the mental wellbeing of crisis line volunteers is important, because studies have shown that volunteers in general are at risk of emotional exhaustion and vicarious trauma, meaning that they are emotionally affected by traumatic life experiences of others (Höing et al., 2016; Howlett & Collins, 2014). Reduced mental wellbeing can also lead to lower quality of care, absenteeism or high turnover (Johnson et al., 2018; Scheepers et al., 2015) which may in the long term endanger crisis line service continuity. However, despite its importance, research on mental wellbeing among crisis line volunteers is scarce. A recent systematic review (Willems et al., 2020) showed that in the past three decades, only thirteen studies on the topic were published. The studies varied strongly in quality, and volunteers’ mental wellbeing was often not the main focus of the study. The review showed that crisis line volunteers seem to experience satisfaction and gratification as a result of their work (Hellman & House, 2006; Praetorius & Machtmes, 2005) but are also at increased risk of impaired mental will being, operationalized as symptoms of burnout (Cyr & Dowrick, 1991; Roche & Ogden, 2017), vicarious traumatization (Dunkley & Whelan, 2006), psychological disorders (McClure et al., 1973), distress (Kitchingman et al., 2016; Mishara & Giroux, 1993), and feelings of frustration and irritation (Pollock et al., 2013; Willems et al., 2020). As far as we know, no studies have been conducted in which volunteers talk from their own perspective about the emotional impact of their work.

Insight into factors associated with mental wellbeing in crisis line volunteers and available resources to deal with these factors is important, because it can inform the development of organizational or personalized interventions aimed at increasing or maintaining the mental wellbeing of volunteers. In their systematic literature review, Willems et al. (2020) also examined which particular factors were associated to the mental well-being of crisis line volunteers. Three categories of factors were established: (a) factors related to the nature of work, such as the subject of the calls and inappropriate behaviour of callers; (b) factors related to the organization, such as supervision, training and co-worker support; and (c) factors related to the volunteer, such as productive or non-productive coping and years of experience. The review also identified some of the resources available to volunteers to deal with the various challenges in their work with the crisis line service, such as training, co-worker support, and regular relaxing activities (Willems et al., 2020). Yet, although these studies have provided some insight into how these factors are associated with the volunteer’s wellbeing, most studies focussed on just a single factor, and a more comprehensive overview of the challenges that crisisline volunteers are facing and the resourses they use to cope with these challenges is missing.

In sum, crisis line services are a valuable addition to regular care, but working at the crisis line can be challenging. In the long term, this may not only reduce the mental wellbeing of the volunteers, but also lead to reduced quality of care, absenteeism or high turn-over rates. Thus far, only few studies have examined the mental wellbeing of crisis line volunteers. The few studies that explored the challenges that volunteers encounter in their work, or the resources they use to cope with these challenges, usually focus on just one single challenge instead of providing an comprehensive overview. With this study we want to address this gap, and aim to provide a comprehensive overview of the emotional impact of the work, challenges, and resources of volunteers working at the crisis line. The main three research questions are: (1) Which positive and negative emotions do crisis line volunteers experience due to the work at the crisis line service? (2) Which challenges related to the nature of their work and the organization contribute to volunteer work-stress? (3) Which personal and work-related resources are helpful in dealing with job-related-stress?

## Methods

The central aim of this study is to understand the emotional impact of volunteering at the crisis line, and to provide a comprehensive overview of the challenges volunteers encounter in their crisis line work, and the resources they use to deal with these. To this aim we use a qualitative, grounded theory approach of which the basis is pragmatism and symbolic interaction. In situations where knowledge is lacking, the Grounded Theory Approach is a suitable method to apply. The aim of a grounded theory approach is to generate new insights rather than to test hypotheses based on existing theory (Charmaz, 2014). To obtain a complete and balanced view, we conducted focus groups with volunteers currently working at a crisis line service, but also interviewed former volunteers who had stopped volunteering at the crisis line service in the past year. By collecting data in the natural environment and by asking open questions, without steering the answers in a certain direction, valuable raw data was collected. From there, inductive and deductive analysis was applied. Ethical approval for the study was obtained from the Ethical Board of the Faculty of Behavioural and Management studies from the University of Twente (no: 17,102).

### Sample

The study was conducted at “the Listen line”, which is a Dutch crisis line service that offers twenty-four hours a day and seven days a week a listening ear to anyone who needs it in case of personal crisis. At the Listen line 1,500 trained volunteers annually hold approximately 330,000 telephone calls, email or chat conversations (Van ’T Foort & Veldkamp, 2019). The current study was conducted in two regions, one in the east of the Netherlands (Zwolle) and one in the west (Rotterdam). Potential focus group respondents were approached by email by the professional trainers of these regions. In the email the purpose of this study was explained and information about the researchers was given (their personal goals (PhD research) and contact details). In total 22 persons consented to participate, and were assigned to one of four focus groups: two in Rotterdam, one with volunteers who had more than three years of experience at the crisis line service (*n*= 8) and the other with less experienced volunteers (*n*= 6) and two focus groups in Zwolle, with five and three participants (based on the availability of the volunteers). In Zwolle experienced and less experienced volunteers were mixed. The age of the participants ranged from 24 to 78 years (mean 57), most were women (77%), and on average they had five years of experience (range 4 months—23 years).

Since it can be expected that those who still volunteer at the crisis line are mainly positive, we also wanted to interview some people who had decided to stop volunteering at the crisis line. In this way we aimed to get a more balanced picture of the perceived emotions, challenges and available resources of volunteers. Therefore, we approached sixteen former crisis line volunteers who gave up volunteering in 2017 for an interview. These former volunteers were selected at random by the headquarters of the Listen line. They were sent an invitation by email with information about the purpose and procedures of the study and an informed consent form. Eight (50%) responded positively and an interview was arranged with them. The age of the participants ranged from 25 to 72 years (mean 57), all were women, and on average they had 4 years of experience (range 1–7,5 years).

This sample mainly corresponds to the total population (gender: female 77%, male 23%; mean age: 57 years; mean number of years of work experience: 6 years) (Székely et al., 2015).

### Procedures and measures

The focus group sessions were conducted on the crisis line service location, in the presence of the respondents and the researchers. They were led by the first author (RCWJW, trained in carrying out focus groups and interviews) and a psychology student of the University of Twente, both of them female, and both had no relationship with the participants in the focus groups. The focus groups lasted about 2 hours, and took place between June 2017 and September 2017. The individual interviews (led by RCWJW and a male psychology student of the University of Twente) were conducted face-to-face (*n*= 2) or via telephone/videocall (*n*= 6). They lasted about 45 minutes. At the start of the focus group and interview, participants were asked to sign an informed consent form. The same topic list was used for the focus groups and the interviews. Participants were first asked to introduce themselves and to briefly describe an experience or a call or chat that had impressed them. Subsequently, three areas were explored: (1) positive and negative emotions, (2) challenges of the work (of the help requests, the caller, the organization, or the volunteers themselves) that can make the work difficult at times, and (3) personal and work-related resources that help in dealing with these challenges (see Table 1 for the focus group and interview schedule).

**Table 1.** Focus group and interview schedule

Topic	Example Questions
1.) What positive and negative emotions did volunteers experience during their work at the crisis line service?	Which positive emotions are you experiencing in your work? Which negative emotions are you experiencing in your work?
2.) What challenges regarding (a) the help requests, (b) the caller, (c) the organization of the crisis line service, or (d) the volunteers themselves do volunteers encounter?	*Focus group*: Silent brainstorm with the use of “post its”. After the brainstorming session, the “post its” were divided over the four categories of factors that make conversation difficult. The “post its” were discussed per category. *Interviews*: Can you give examples of conversations that were difficult for you? Are there any personal characteristics that make the work more difficult for you/some people than for others?
3.) Which resources can facilitate coping with difficult conversations or other challenges?	What kind of thoughts or behaviours are helpful during or after a conversation? How does the organization help you to deal with challenges?

### Data analysis

The focus groups and interviews were audiotaped with the prior consent of all participants, and transcribed verbatim. Transcripts of all focus groups and interviews were edited to remove any personal information which could identify the respondent and were uploaded into Atlas-Ti data analysis software.

The data of the interviews and focus groups were analysed by two independent coders (RCWJW and CHCD), using open, axial, and selective coding, and the method of constant comparison.

Data collection and analysis were carried out simultaneously, which means that as soon as the first focus group interview was transcribed, coding took place immediately. Both coders separately read the transcripts of the first two focus groups. Based on these two focus groups, the coders developed a first provisory coding scheme, using inductive coding (open coding). Inductive analysis helps to structure raw data into a summary format and to establish links to the research objectives (Charmaz, 2014). After two focus group interviews, themes and the relationships between themes, were identified. On the basis of these themes other focus group interviews and individual interviews were analysed using inductive analysis (axial coding). After the inductive analysis, deductive analysis was applied by selective coding on the basis of previously defined themes (selective coding). Based on this, final themes were determined. The coders discussed the differences in identified themes to fully reach consensus, previously found themes were verified and deepened and subthemes were identified until saturation was reached. This final coding scheme was also used in the analysis of the interviews. At the end of the analysis process, all transcripts from the focus groups and individual interviews were read again and compared with the code tree to ensure that no important information had been overlooked.

Because the themes from the focus group interviews and the individual interviews matched, the results will not be presented separately. Extra themes that arose from the individual interviews will be mentioned. The analyses were conducted in our native language (Dutch). Codes and themes were checked and discussed with a native English speaker. De citations were translated from Dutch into English by a native English speaker, to ensure translation validity.

## Results

**Figure 1.**
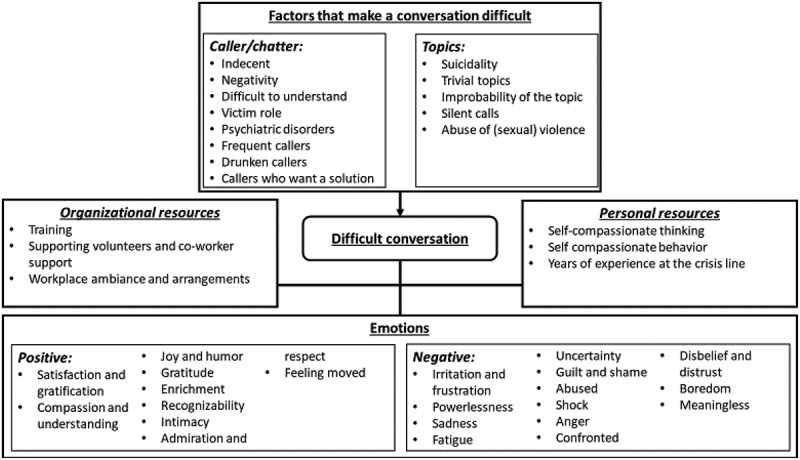
Schematic representation (coding tree) of the results

The results are described on the basis of the research questions. The order in which the results are described is from most frequently mentioned to the least frequently mentioned emotions, challenges and resources. Figure 1 shows a schematic representation (code tree) of the results of the interview.

### Emotions due to the crisis line work

In this section we present volunteers’ positive and negative emotions.

#### Positive emotions

Participants met a wide range of positive emotions and experiences.

*Satisfaction* was the most frequently mentioned positive emotion. The feeling of satisfaction that volunteers experienced resulted from the realization that the work they do makes a difference in the lives of callers. Volunteers did not always have to fully solve the problems of callers, but to help someone get started on therapy, or to guide someone in the last moments before his death, was experienced as satisfying.

*“That conversation lasted a long time and he was very happy that he was able to express this for the first time, because he thereby also drew strength from it so that he could go see a counselor.”*

In addition, volunteers also mentioned feeling of satisfaction with their intervention during a call and the positive impact this intervention had on the callers, expressed by their gratitude.

Volunteers identified *compassion and understanding* as a positive emotion, because these skills connected them emotionally with callers, which motivated them to alleviate the suffering of callers.

*“Then I spoke to a 16-year-old girl who called in the middle of the night, she was in the middle of her 6th chemo treatment. She felt lonely, while her sisters were chatting, and she was shivering from the cold and misery. And then I felt a lot of compassion”*

Volunteers mentioned *joy and humour* as essential emotions to alleviate the heaviness of the conversations. Humour was deliberately used by volunteers to lighten a complex conversation. Many volunteers were very alert to a joke or a laugh from the caller, because they recognized it as the turning point in a conversation.

*“There you get so much depth, you can reach people in this way, by going out of the serious mode, and going into depth with humor. Then everyone is incredibly grateful, because then they open up.”*

By listening to the stories of callers and the circumstances in which they found themselves, volunteers experienced *gratitude* for the favourable conditions in which they themselves lived, but they also experienced gratitude for the enrichment that came from deep conversations and the feeling of being able to contribute.

*“And then I realize that there is so much suffering, so much suffering. And that I may consider myself lucky that I lead a happy life.”*

Volunteers experienced *enrichment* as positive emotion, because they learned a lot from the conversations, for example, communication skills, acceptance that callers’ behaviour cannot always be influenced by the volunteer, or the ways in which they could handle difficult situations.

*“If a call is not going smoothly, there is enough reason to see how you did it and then ask for feedback on what to improve next time. I find that very enjoyable.”*

Other positive emotions were recognizability, intimacy, admiration/respect, and feeling moved. Volunteers felt comfortable with difficult conversations when they *recognized* the callers’ story; they mentioned a positive feeling because they could respond correctly based on their own experience. Volunteers experienced great *intimacy* and involvement during conversations. Some volunteers said that they felt pride that the callers chose them to share their experiences. Volunteers felt *admiration/respect* for the way callers managed to survive despite their problems.

### Negative emotions

Participants also met a wide range of negative emotions and experiences:

*Irritation and frustration* were often experienced, mostly by callers who manipulated and provoked volunteers; callers who were critical or accusatory to volunteers or the crisis line service, and callers who tried to shock by, for example, opening the conversation with a suicide threat.

*“Or a caller who says (cheerfully): ‘Well, then I’ll just have to put an end to my life’. (… .) Yes, some callers only really want to shock. Well, I do not like that.”*

Irritation and frustration were also provoked by passive callers and callers who blamed everybody else but themselves, who put the responsibility for the course of the conversation on the volunteer, and stayed in the victim role. The slowness of the caller who wanted to tell in detail what he had done triggered irritation, as well as people who called to make fake calls, sex callers and drunken callers.

*“I find these conversations senseless and certainly drunk callers who, when they are sober, no longer know that they have called. Why call now, it doesn’t make any sense.”*

The frequent caller (the caller who calls several times a day, often with the same story), people who complain, whine, tend to think in extremes and do not want to listen to tips and advice from the volunteer were also mentioned as irritating and frustrating.

*“But on the phone, you have frequent callers whose voice I recognize. And if the person actually has the same story as last time and there has been no progress at all, I find that quite difficult.”*

Finally, irritation was generated if the caller did something else during the conversation, for example, washing dishes or watching TV, while the volunteer was concentrating on the conversation.

The feeling of *powerlessness* often arose when the volunteer was not able to do something for the caller, for example, giving a hug when someone is having a hard time or wanting to solve the problem for someone.

*“People who really suffer, like Sunday night, I had a lady on the phone, who was really depressed. Yes, you can come up with anything, but I just know that it won’t help. I find that so difficult.”*

Powerlessness also arose from the position in which callers found themselves because of the effects of social policy, in particular cutbacks at mental health organizations.

*“People who have to live on their own with the current healthcare. Independently as long as possible. And then they have to do with one and a half hour of care once a week, … .”*

Another aspect that caused powerlessness was the fact that volunteers could not do anything because callers call anonymously. In addition, the crisis line service applies the philosophy of non-intervention, this means that volunteers are only supposed to offer a listening ear and will not intervene therapeutically.

Volunteers feel *sadness* about the suffering that the callers experienced. Sometimes they switched off their phone for a moment and then cried because of the distress of the caller. There was also sadness about the suicidality of callers.

*“And I recently had a conversation with a girl, a woman, in her early 20s, who lost her whole family. It stayed with me and it was also the first time I was actually crying during the chat.”*

*Fatigue* came mainly from callers who tell the same thing over and over again, people who chatter without the volunteer being able to get a word in, people who do not say a word, frequent callers, and callers who expect an immediate solution to their problems.

*“(… .) but with some people you just know, they just call four times in a row. Within an hour, and they will just tell you exactly the same. You hear that sometimes, because you speak to your colleague in the room next door, who says: Gosh, I just had that gentleman on the phone.”*

Volunteers mentioned feelings of *guilt and shame*, because they made mistakes, felt no connection, or could not help the callers. Shame was caused by making promises to the caller that volunteers could not fulfill.

Volunteers experienced *uncertainty* about their skills, especially when discussing suicide or when there was no connection with the caller. Uncertainty about the outcome of a call (how things ended with a caller) was also mentioned.

*“You have a conversation with someone and they will disappear from the screen once the conversation has ended. And you don’t know how it will go afterwards. There is no follow-up unless you get the same person on the chat again or on the phone.”*

*Anger* was provoked when the caller shouted at volunteers, had an aggressive tone, or called to gain sexual gratification.

*“I have been angry. Especially on people who scolded me or crossed the border in some other way. People who called for sexual gratification. Well that’s my limit.”*

Anger was also generated when the volunteer heard that the caller was being misjudged, or when children or animals were involved.

Other negative emotions and experiences were abused, shocked, confronted, disbelief and distrust. Volunteers felt *abused* when they noticed too late that they had a sex caller on the phone, when the caller used them as a wailing wall, or when callers were judgemental or discriminating. *Shock* was felt when callers were angry and scold, if the caller had a suicide wish, or callers told weird stories. Volunteers experienced a conversation as *confrontational* when it evoked memories of painful events which the volunteers have experienced themselves. *Disbelief and distrust* were experienced when volunteers doubted the stories that callers had because they were so weird or implausible.

Two other negative emotions that emerged during the interviews were boredom and meaninglessness. Volunteers felt *boredom* when they had to carry the same type of conversation over and over again, and then failed to keep their attention in the conversation. *Meaninglessness* was felt when the caller did not benefit from the conversation, or when the ability to call keeps the caller from seeking professional help.

*“ … . but I don’t have to go looking for contacts either, because I can always call the Listen Line, so then you were also a factor for them to no longer challenge themselves to do anything.”*

## Challenges that make the work difficult at times: job stressors

There were different challenges volunteers met, that make their work difficult at times. Two types of stressors were identified from the focus groups and the interviews: caller/chatter-related stressors and topic-related stressors.

### Caller-related stressors

The most frequently mentioned distressing characteristic of the caller was *indecency*. Volunteers found it offending when callers criticized the crisis line service or colleagues, when they were insulting, scolded the volunteer, making discriminating remarks, and manipulated the volunteer.

*“Then you think, we are all volunteers, we all do our best and you are only complaining about us. That does not feel good.”*

Some volunteers did not know how to respond to sexually abusive calls and felt abused when they noticed that a caller was masturbating during the conversation.

A distressing characteristic of the caller was *negativity*. Volunteers found it difficult to connect with callers who were negative and did not listen to the volunteer, callers who only wanted to complain, who kept rattling without the volunteer being able to cut in, or callers who barely made contact.

Sometimes callers were *difficult to understand*, this could be related to dialect, drunkenness, aphasia, stuttering or other speech impediments. It could also be due to the slowness or speed at which the caller spoke, or overly difficult language.

*“(…) after the umpteenth monologue, after a story that I barely understood, I do not know what it is about, but just leave it, just for the sake of simplicity, let the person tell his story. I got distracted at one point, so that the one on the other side said “hey, are you still there?”*

The caller in the *victim role* was experienced as a stressor. These callers were passive, blamed their problems on others, and had no self-reflection. They did not accept anything from the volunteer and refused to reflect on their own behaviour.

*Psychiatric symptoms*, such as hyperactivity, slowness, hallucinations, and delusions could disrupted the contact between the caller and the volunteer. Volunteers felt powerlessness towards callers, because of callers’ illness.

*“Then a caller with a bipolar disorder or something related and who is a very intelligent person who sees himself empty-handed. He just knows that there is a disturbance. I mean, those people are not crazy.”*

Other characteristics of callers that could make a conversation difficult were frequent callers, drunken callers, callers who wanted a solution, or callers who saw the volunteer as their friend. *Frequent callers*, who called multiple times with the same story. Volunteers experienced *drunken callers* as difficult. Often these conversations were difficult to follow and perceived as meaningless, because volunteers doubted if the caller would remember the conversation when they were sober again. Volunteers mentioned callers who were *doing other things during the conversation*, such as washing dishes or watching television as difficult, because it distracted them from the conversation. Callers sometimes became angry or disappointed when they did not *get a solution*.

### Topic-related stressors

The most frequently mentioned topic that puts a lot of strain on the volunteers’ concentration was *suicidality*, when callers indicated that they wanted to end their lives. Conversations on this subject were experienced as heavy and intense.

Volunteers found it also stressful if the topics were too *trivial* or if callers called just to be funny.

*“And calling just to be calling. ‘Hello Netherlands.’ Who just want to be funny. That’s how I characterize it. It is a large group, that offends me.”*

*Improbably of the topic* was experienced as inconvenient. Sometimes volunteers doubted the truth of the stories of callers, thereby they could not make a correct assessment of the caller.

*“(… .) people who tell a beautiful story that you feel, than you think, this is not entirely true. (… .) Then you ask a question, and then they hang up. Then you know that your feeling is correct.”*

Other topics volunteers found stressful were silent calls and abuse. *Silent calls*, when callers who did not say anything, or placed the responsibility for the course of conversation with the volunteers. A number of volunteers had trouble with conversations about *abuse*, in particular child and animal abuse.

## Resources for dealing with challenges

Finally we asked the volunteers what helped them to deal with these challenges. The answers could be divided into personal and organizational resources and are discussed below. In addition, also examples will be discussed if volunteers do not experience this resource.

### Personal resources

The personal resources could be divided into self-compassionate thinking, self-compassionate behaviour, and years of experience at the crisis line. Many volunteers had *self-compassionate thinking* by being aware that there was a mutual responsibility for the course of the conversation. They dared to admit their emotions, allowed themselves to make mistakes, and knew how to let go of the content of the conversations. The anonymity helped them to let go.

*“When the last call is over, it is done. It’s good to be involved, to show empathy and things like that, but at the end of the day, it’s what it is, you have no control over it.”*

Some volunteers tend to be more self-critical than self-compassionate. For example, being ashamed that they had said something that they could not fulfill, being angry when they made mistakes, ruminating on difficult conversations or chats, or being too strict for themselves.

*“And then I worry all weekend about it. At the training it all came out. Yes, that was stupid of me. I thought I had dealt with it, but I hadn’t. I thought that was stupid of myself.”*

*Self-compassionate behaviour* manifested itself by taking timely breaks, setting boundaries for the caller and the organization, or seeking contact with co-volunteers or trainers. Some volunteers were taking breaks in order to process difficult conversations, by switching off the phone or they ate or drank something, went outside or to the toilet, cried or prayed, or made a report of the conversation. In order to bring the shift to a good end before they picked up their own lives, many volunteers liked to cycle, perform rituals or make an extensive report about the service.

*“After a difficult conversation, it is nice to leave the computer for a while and do something else, if only to make a cup of tea, something like that, that is yes, that is nice to do.”*

There were also volunteers who did not take a break because they forgot to switch off the phone. Other volunteers saw that the lines were busy and, therefore, did not want to take a break. Starting volunteers preferred not to take a break because they wanted to prove themselves. Many volunteers were able to specify limits to the caller. If a caller started to scream or did not want anything, if the subject triggered the volunteer, or if the caller was drunk or a sex caller, the volunteer ended the conversation. Volunteers also indicated their limits by making it clear that they felt uncomfortable with the conversation, for example, if they felt irritated or when there was no connection between the caller and the volunteer.

*“Then I try to refrain in a friendly quiet way from talking to long and end the conversation. ‘Sleep it off or go and drink a cup of coffee’.”*

Sometimes it was difficult for volunteers to end a conversation, the volunteer spent too much time listening and became curt with the caller. Volunteers sometimes gave too much information about themselves during a conversation.

*“I am honest and open and I do not mind sharing something, but at some point they will be in my personal space and I really want to protect that space and that is what happened all the time.”*

Seeking contact with the trainers or with family (if they worked from home) when a conversation was difficult is another example of self-compassionate behaviour. When they worked at the location of the crisis line service, fellow volunteers often also had a chat with each other.

*“There is always the possibility to call someone from the management of the crisis line service, but in most cases that is not necessary at all, if you can talk to each other about it when you are having a hard time.”*

Sometimes volunteers did not have the opportunity to contact colleagues because they worked from home. Volunteers did not want to bother the backguard and, therefore, did not seek help for their problem.

Specify limits to the crisis line service was reported as a protecting factor. When a shift was almost over, or if the shift had been heavy, some volunteers choose to not answer a telephone call or a chat. Some volunteers refused to conduct talks from the suicide prevention line.

*“I mean ‘suicide prevention’ is very pro-life. Then I think, those people have tried everything, had all kinds of therapies and conversations. All at a dead end. And I’ll never say ‘boy, just put an end to it’. But I can imagine it very well.”*

Volunteers could see on the computer screen whether or not all lines were occupied. A few volunteers indicated that they did not logoff after a heavy conversation because they saw that all lines were occupied.

Volunteers mentioned that years of *experience at the crisis line* and having more skills, could help them to deal better with their own emotions. Volunteers with life experience stated that they could show more understanding for the situation the caller was in.

Less experienced volunteers had fewer opportunities to respond to difficult questions.

*“I have also had a conversation about suicide, and I thought: did I handle it well? Not that mistakes were made, but perhaps that someone who had more experience at that moment had a bit more to offer.”*

### Organizational resources

The *training* and courses organized by the crisis line service were experienced as positive, in particular the intervision, where experiences were shared with co-workers under supervision.

*“During the intervision a caller is sometimes discussed, about whom others say: ‘oh, I also had that caller on the phone, I handled it very differently’. And the way he tells it, I think oh, that’s the way I should actually do it in the future.”*

Some volunteers said the courses did not add anything. This was mainly related to the suicide prevention training. There were also volunteers who found this training too confronting.

The *supporting of volunteers and co-worker support* was perceived by volunteers as an important resource. When volunteers had heavy conversations, there was always the possibility to contact the crisis line service. The trainer, together with the volunteer, assessed whether extra care was needed. If the reports showed that conversations had made a great impression on the volunteer, the trainer from the crisis line service would contact the volunteer to see how things were going.

*“Well, they called me, to ask and see what it had done to me. To be certain that my well-being was fine.”*

Co worker support was felt by volunteers to be an important form of support. It gives them the opportunity to discuss what has happened during their service and to reflect together on a conversation.

*Workplace ambiance and arrangements* were experienced as resources. Volunteers experienced the ambiance and culture at the crisis line service as warm, open, safe, and confidential. Telephone shifts of four hours were sometimes experienced as too long, especially when there were heavy conversations that required a lot of concentration. Participants in our study experienced no problems with nightshifts, but they mentioned that a colleague stopped volunteering, because the nightshifts were too burdensome. The crisis line service offered volunteers a *workplace*, but there was also the possibility to work from home. Some volunteers wanted a clear distinction between work and their private life, while other volunteers were glad that they could work at home. When volunteers choose to work at home, the volunteers who worked on location missed the contact with colleagues and the co-worker support.

*“At the weekend there is no one here, you are alone in the building. You can never tell your story, there are no sparring partners.”*

Volunteers sometimes experienced hindrance from the environment, such as the location from where volunteers have to do their work, background noise, too quiet, and too high or too low a temperature.

*“But if there is a course going on here or something else during my telephone shift, I am quite curious by nature, then I want to know who is there and then I get distracted.”*

The crisis line service offers sufficient technical support at the workshop, but also at home. Technical malfunctions can be disrupting. Sometimes a chat conversation ended because of technical faults. Disruption to the telephone caused the crisis line service to be less accessible to people with problems. Volunteers experienced this as difficult because they want to be there for the callers.

## Discussion

This is the first study that comprehensively examined crisis line volunteers’ positive and negative emotions during their work, the challenges they experience in their work and the available resources they use to cope with these challenges.

Our study reveals that whereas many volunteers experience positive emotions in their work, such as gratification, compassion, gratitude and joy, they also mentioned a wide range of negative emotions. Most common were irritation and frustration, provoked by inappropriate, manipulative, or passive callers. Frequent callers and fake callers also provoked this emotion in many volunteers. The emotions of fatigue, uncertainty, abuse, anger, and feelings of disbelief and distrust are clearly linked to these types of callers. Shame and guilt arise when volunteers have the feeling that they do not respond adequately to these callers. All these negative emotions were also described in the study of Pollock et al. (2013). A number of negative emotions were not mentioned earlier in previous research, such as the powerlessness that arises when volunteers are not able to give callers what they need, such as a hug, or when callers indicate that they are suffering because of the cutbacks in elderly care and mental health care. Also, the sadness that volunteers feel when a story of a caller touches them, or when they find recognition in a story of the caller are emotions that have not been described before. Two striking negative emotions, namely boredom during a conversation and a feeling of meaninglessness, were mentioned during the interviews with former volunteers. It is important to pay attention to these negative emotions during training and supervision, because negative emotions could lead to higher exhaustion in work, with higher turnover intentions as result (Chau et al., 2009).

This study identified a wide range of caller-related challenges faced by volunteers in their work: callers who show inappropriate behaviour (such as scolding, being manipulative, (sexually) abusive or remain silent), callers who are difficult to understand, have psychiatric problems, are drunk, call frequently, or seem to make up stories. Similar results were found by Pollock et al. (2013). In order to help volunteers handle these difficult calls and avoid too much involvement, the principle of “non-disclosure” is often recommended, which means that volunteers are not allowed to share anything of their identity or personal experiences with the caller (Pollock et al., 2013). However, sharing of personal experiences could be helpful to connect with the callers; it could be used to demonstrate understanding of the caller’s situation, and compensate for the anonymous and disembodied nature of helpline contact (Mishara et al., 2007). In the past, interventions have been developed to help volunteers to better deal with sexually abusive calls (Baird et al., 1994; Matek, 1980) and frequent callers (Barmann, 1980; Brunet et al., 1994; Hall & Schlosar, 1995). Unfortunately, these interventions are dated and have not been tested for effectiveness. As long as there are no effective interventions to deal with inappropriate callers and frequent callers, it is important that during training attention is paid to handling challenging callers and discuss the limit in sharing personal experiences with the callers.

Caller/topic related challenges that have not been described in empirical research before, are: negativity of callers, callers in a victim role, demanding callers who want immediate solutions for their problems, and (too) trivial topics of conversation. These stressors are challenging because volunteers have difficulties in feeling connection with callers who are only negative or who remain in a victim role. These callers often do not want to listen or reject any suggestion or advice from the volunteer, or tend to externalize all of their problems. Also, callers who want immediate solutions for their problems are challenging for the volunteer, because something is expected that they cannot offer. Callers who call with trivial issues can make the volunteer feel that they should actually spend their time better on people with “real” problems. Future research should address these challenges and examine how often they occur and to what extent they influence the volunteers’ mental wellbeing.

Volunteers mentioned several resources to cope with negative emotions and experiences. First, positive emotions can help crisis line volunteers dealing with or balancing work-related stressors. In this study volunteers mentioned a wide range of positive emotions. Most frequently mentioned were gratification, compassion, joy/humour, gratitude, and enrichment. Positive emotions are important because these emotions generate more flexibility, more creativity, and play an important role in bouncing back from negative emotions (Fredrickson, 2001; Fredrickson & Joiner, 2018; Tugade & Fredrickson, 2004, 2007). Positive emotions are also associated with increased self-efficacy, self-esteem, and optimism (Xanthopoulou et al., 2012). Second, self-compassionate thinking (e.g., admitting emotions, allowing themselves to make mistakes) and self-compassionate behaviour (e.g., taking breaks, seeking contact with co-workers) were mentioned as useful coping resources. There is a growing body of evidence that self-compassion decreases negative emotions, such as fear, anger, and shame (E. A. Johnson & O’Brien, 2013; Neff & Vonk, 2009) and increases self-reassurance (Sommers-Spijkerman et al., 2018), positive emotions, such as happiness, joy, and wellbeing (Baer et al., 2012; Trompetter et al., 2017). However, as far as we know the role of self-compassion has not been examined in crisis line volunteers. More research on this topic is needed, because especially for this highly challenged group, self-compassion could be essential to buffer the impact of work-related stressors on mental wellbeing. Future interventions could try to enhance these personal resources by encouraging volunteers to focus on positive emotions and by cultivating self-compassion.

Our findings also underscored the importance of organization-related resources such as training, supporting volunteers and co-worker support, and workplace ambiance and arrangements. Many researchers have emphasized the importance of good training and guidance of crisis line volunteers (Cyr & Dowrick, 1991; Dunkley & Whelan, 2006; Hellman & House, 2006; Sundram et al., 2018; Yanay & Yanay, 2008). At the Dutch “Listen Line”, volunteers are obliged to follow a training, which consists of an e-learning part and various meetings in which mainly conversations are practiced. During the training potential volunteers will be supervised by experienced volunteers. In addition, regular meetings are organized with a certain theme (e.g., listening to people with a psychiatric disorder). Once a year, the motivation and well-being of the volunteer is discussed with a professional trainer. A professional trainer is available day and night for the volunteer who is in great need of guidance after a telephone conversation (Van ’T Foort & Veldkamp, 2020). In our study the participants were very positive on training and guidance they receive from the organization. It is recommended that during the selection process potential volunteers are made aware that this work can have an emotional impact and that the volunteer will face a wide range of challenges. It is also recommended that the requirements that volunteers need in order to cope well with the challenges in the work at the crisis line service, are determined in advance.

Co-worker support was also mentioned as an important resource for handling work challenges. A number of volunteers reported a lack of co-worker support. Lack of social support was also mentioned in previous research (Cyr & Dowrick, 1991; Hellman & House, 2006; Sundram et al., 2018). Crisis line organizations should facilitate contact between co-workers, because research has shown that co-worker support can protect from depression, buffer the effect of job demands on workplace stress (Kitaoka-Higashiguchi et al., 2003), and generate a sense of belonging to a group with a common goal, which contributes to satisfaction and has a positive influence on mental well-being (Triandis, 2001). In the case of crisis line volunteers, contact with co-workers is often hampered because many volunteers choose to work from home. In these cases the organization should search for alternatives for co-worker support, such as online coffee breaks or regular joined activities to facilitate bonding.

Workplace ambiance and arrangements, such as length of shifts and environment, were mentioned as organizational resources. This study also found that the length of the shifts (mostly four hours) was sometimes perceived as too long, especially since some telephone calls require a lot of concentration and are therefore exhausting. In order to keep volunteers motivated and healthy, the crisis line organization could explore the possibility of creating more personalized schedules. Finally, the environment (e.g., noise or unpleasant location of the office) and technical malfunctions (electricity failure) are perceived as challenging. By offering volunteers the choice to work either from home or from the office, the problem of an unpleasant environment can be overcome. Fortunately, technical malfunctions did not occur very often. In addition, volunteers emphasized the importance of a good ambiance, which is encouraged by organizing regular informal meetings, for example, a barbeque or an excursion once a year. Volunteers in this research indicate that these are resources that make the work more enjoyable and enriching for them.

There are a number of strengths of this study. First, by using a qualitative approach we obtained insight in a broad range of emotions, challenges, and resources that are important in the wellbeing of crisis line volunteers. It provides a comprehensive overview of relevant factors and gives input for specific interventions and policies aimed at increasing mental wellbeing in crisis line volunteers and reducing turn-over rates. Second, by including not only active volunteers, but also former volunteers we obtained a complete overview of experiences and views. Future research is needed to get insight into how often particular stressors occur and their perceived levels of stress and their relationship to mental well-being. For this reason we are currently constructing a questionnaire to quantitively assess the occurrence and perceived stressfulness of the caller- and organization related stressors mentioned in this study. Yet, the study has also some limitations. The first is the possibility of selection bias, because potential respondents were approached by their trainers and therefore not all volunteers had an equal opportunity to participate. Second, the method of focus groups has the risk that quiet respondents can be overwhelmed by the more verbally skilled. To ensure that everybody’s voice was heard, the focus group contained a part in which individuals were asked to write down their opinions/experiences first on post-its, before discussing them in the group. Moreover, a second interviewer was present alongside the main interviewer, to intervene if some respondents were talking too much. The methodology of a focus group also has an important advantage: because the respondents in a focus group question each other and discuss the subject, deeper insights are obtained (Evers, 2015).

To conclude, this is the first study that provides a comprehensive overview, based on the perspective of volunteers, of the emotional impact of work on the crisis line, the challenges volunteers encounter and the resources they use to deal with them. This study highlights the importance of (1) training of volunteers in dealing with various specific types of callers such as inappropriate callers or callers with psychiatric symptoms, and (2) personalized policies to support motivation and job satisfaction of volunteers. In addition, this study provides valuable input for the development of interventions aimed at increasing personal resources, such as awareness of positive emotions and self-compassion. These personal resources can help to increase the mental wellbeing of crisis line volunteers and reduce turn-over rates. Volunteers with a high degree of mental wellbeing are fundamental to the continuity of the crisis line service.

## References

[cit0001] Aguirre, R. T. P., & Bolton, K. M. W. (2013). Why do they do it? A qualitative interpretive meta-synthesis of crisis volunteers’ motivations. *Social Work Research*, 37 (4), 327–13. 10.1093/swr/svt035

[cit0002] Baer, R. A., Lykins, E. L. B., & Peters, J. R. (2012). Mindfulness and self-compassion as predictors of psychological wellbeing in long-term meditators and matched nonmeditators. *The Journal of Positive Psychology*, 7(3), 230–238. 10.1080/17439760.2012.674548

[cit0003] Baird, B. N., Bossett, S. B., & Smith, B. J. (1994). A new technique for handling sexually abusive calls to telephone crisis lines. *Community Mental Health Journal*, 30(1), 55–60. 10.1007/BF02188875 8149723

[cit0004] Barmann, B. C. (1980). Therapeutic management of chronic callers to a suicide prevention center. *Journal of Community Psychology*, 8(1), 45–48. 10.1002/1520-6629(198001)8:1<45::AID-JCOP2290080108>3.0.CO;2-8

[cit0005] Brunet, A. F., Lemay, L., & Belliveau, G. (1994). Correspondence as adjunct to crisisline intervention in a suicide prevention center. *Crisis*, 15 (2), 65–68. 76 7988167

[cit0006] Charmaz, K. (2014). *Constructing grounded theory* (2 ed.). Athenaeum Uitgeverij.

[cit0007] Chau, S. L., Dahling, J. J., Levy, P. E., & Diefendorff, J. M. (2009). A predictive study of emotional labor and turnover. *Journal of Organizational Behavior*, 30(8), 1151–1163. 10.1002/job.617

[cit0008] Cyr C., & Dowrick, P. W. (1991). Burnout in crisisline volunteers. *Administration and Policy in Mental Health*, 18(5), 343–354. 10.1007/BF00707000

[cit0009] Dunkley, J., & Whelan, T. A. (2006). Vicarious traumatization in telephone counsellors: Internal and external influences. *British Journal of Guidance and Counseling*, 34(4), 451–469. 10.1080/03069880600942574

[cit0010] Evers, J. (2015). Kwalitatief onderzoek, een korte inleiding. In *Kwalitatief interviewen: Kunst én kunde*(2 ed., pp. 14–15). Boom Lemma Uitgevers.

[cit0011] Fredrickson, B. L., & Joiner, T. (2018). Reflections on positive emotions and upward spirals. *Perspectives on Psychological Science*, 13(2), 194–199. 10.1177/1745691617692106 29592643 PMC5877808

[cit0012] Fredrickson, B. L. (2001). The role of positive emotions in positive psychology: The broaden-and-build theory of positive emotions. *American Psychologist*, 56(3), 218–226. 10.1037/0003-066X.56.3.218 PMC3122271 11315248

[cit0013] Gould, M. S., Kalafat, J., Harrismunfakh, J. L., & Kleinman, M. (2007). An evaluation of crisis hotline outcomes. Part 2: Suicidal callers. *Suicide & Life-threatening Behavior*, 37(3), 338–352. 10.1521/suli.2007.37.3.338 17579545

[cit0014] Hall, B., & Schlosar, H. (1995). Repeat Callers and the Samaritan Telephone Crisis Line–a Canadian Experience.*Crisis* 16(2), 66–71, 89. 10.1027/0227-5910.16.2.66 7587293

[cit0015] Hellman, C. M., & House, D. (2006). Volunteers serving victims of sexual assault. *The Journal of Social Psychology*, 146(1), 117–123. 10.3200/socp.146.1.117-123 16480125

[cit0016] Höing, M., Bogaerts, S., & Vogelvang, B. (2016). Helping sex offenders to desist offending: The gains and drains for coSA volunteers—A review of the Literature. *Sexual Abuse: Journal of Research and Treatment*, 28(5), 364–402. 10.1177/1079063214535816 24906364

[cit0017] Howlett, S. L., & Collins, A. (2014). Vicarious traumatisation: Risk and resilience among crisis support volunteers in a community organisation. *South African Journal of Psychology*, 44(2), 180–190. 10.1177/0081246314524387

[cit0018] IFOTES. (2020). IFOTES: International Federation of Telephone Emergency Serices. https://www.ifotes.org/en/about

[cit0019] Johnson, E. A., & O’Brien, K. A. (2013). Self-compassion soothes the savage ego-threat system: Effects on negative affect, shame, rumination, and depressive symptoms. *Journal of Social and Clinical Psychology*, 32(9), 939–963. 10.1521/jscp.2013.32.9.939

[cit0020] Johnson, J., Hall, L. H., Berzins, K., Baker, J., Melling, K., & Thompson, C. (2018). Mental healthcare staff well‐being and burnout: A narrative review of trends, causes, implications, and recommendations for future interventions. *International Journal of Mental Health Nursing*, 27(1), 20–32. 10.1111/inm.12416 29243348

[cit0021] Kalafat, J., Gould, M. S., Munfakh, J. L., & Kleinman, M. (2007). An evaluation of crisis hotline outcomes Part 1: Nonsuicidal crisis callers. *Suicide and Life-Threatening Behavior*, 37(3), 322–337. 10.1521/suli.2007.37.3.322 17579544

[cit0022] King, R., Nurcombe, B., Bickman, L., Hides, L., & Reid, W.(2003). Telephone counselling for adolescent suicide prevention: Changes in suicidality and mental state from beginning to end of a counselling session. *Suicide & Life-threatening Behavior*, 33(4), 400–411. 10.1521/suli.33.4.400.25235 14695055

[cit0023] Kitaoka-Higashiguchi, K., Nakawaga, H., Morikawa, Y., Ishizaki, M., Miura, K., Naruse, Y., … Sukigara, M.(2003). Social support and individual styles of coping in the Japanese workplace: An occupational stress model by structural equation analysis. *Stress and Health: Journal of the International Society for the Investigation of Stress*, 19(1), 37–43. 10.1002/smi.953

[cit0024] Kitchingman, T., Wilson, C., Caputi, P., Wilson, I., & Woodward, A.(2016). Testing a model of functional impairment in telephone crisis support workers. *Crisis*, 1–10. 10.1027/0227-5910/a000435 27869506

[cit0025] Matek, O.(1980). Teaching volunteers of a crisis phone service to respond therapeutically to callers with sex problems. *Journal of Sex Education and Therapy*, 6(2), 24–28. 10.1080/01614576.1980.11074672

[cit0026] McClure,J., Wetzel,R., Flanagan,T., McCabe,M., & Murphy,G.(1973). Volunteers in a suicide prevention service. *Journal of Community Psychology*, 1(4), 397–398. 10.1002/1520-6629(197310)1:4<397::AID-JCOP2290010413>3.0.CO;2-4

[cit0027] Mishara, B. L., Chagnon, F., Daigle, M., Balan, B., Raymond, S., Marcoux, I., & Berman, A.(2007). Which helper behaviors and intervention styles are related to better short-term outcomes in telephone crisis intervention? Results from a silent monitoring study of calls to the U.S. 1–800-SUICIDE network. *Suicide & Life-threatening Behavior*, 37(3), 308–321. 10.1521/suli.2007.37.3.308 17579543

[cit0028] Mishara, B. L., & Giroux, G.(1993). The relationship between coping strategies and perceived stress in telephone intervention volunteers at a suicide prevention center. *Suicide & Life-threatening Behavior*, 23(3), 221–229. 10.1111/j.1943-278X.1993.tb00181.x 8249033

[cit0029] Neff, K. D., & Vonk, R. (2009). Self-compassion versus global self-esteem: Two different ways of relating to oneself. *Journal of Personality*, 77(1), 23–50. 10.1111/j.1467-6494.2008.00537.x 19076996

[cit0030] Pirkis, J., Middleton, A., Bassilios, B., Harris, M., Spittal, M. J., Fedszyn, I., … Gunn, J. (2016). Frequent callers to telephone helplines: New evidence and a new service model. *International Journal of Mental Health Systems*, 10(1 1–9). 10.1186/S13033-016-0076-4 27247615 PMC4886390

[cit0031] Pollock, K., Moore, J., Coveney, C., & Armstrong, S. (2013). Configuring the caller in ambiguous encounters: Volunteer handling of calls to Samaritans emotional support services. *Communication & Medicine*, 9(2), 113–123. 10.1558/cam.v9i2.113 24498696

[cit0032] Praetorius, R., & Machtmes, K. (2005). Volunteer crisis hotline counselors: An expression of spirituality. *Social Work and Christianity*, 32(2), 116–132.

[cit0033] Roche, A., & Ogden, J. (2017). Predictors of burnout and health status in Samaritans’ listening volunteers. *Psychology, Health & Medicine*, 22(10), 1169–1174. 10.1080/13548506.2017.1280176 28076965

[cit0034] Scheepers, R. A., Boerebach, B. C. M., Arah, O. A., Heineman, M. J., & Lombarts, K. M. J. M. H. (2015). A systematic review of the impact of physicians’ occupational well-being on the quality of patient care. *International Journal of Behavioral Medicine*, 22(6), 683–698. 10.1007/s12529-015-9473-3 25733349 PMC4642595

[cit0035] Sommers-Spijkerman, M., Trompetter, H., Schreurs, K., & Bohlmeijer, E. (2018). Pathways to improving mental health in compassion-focused therapy: Self-reassurance, self-criticism and affect as mediators of change. *Frontiers in Psychology*, 9 1–12 . 10.3389/fpsyg.2018.02442 30568617 PMC6290051

[cit0036] Sundram, F., Corattur, T., Dong, C., & Zhong, K. (2018). Motivations, expectations and experiences in being a mental health helplines volunteer. *International Journal Of Environmental Research And Public Health*, 15(10), 2123. 10.3390/ijerph15102123 PMC6210510 30261682

[cit0037] Székely, A., Hal, M., Tóth, M. D., & Ádám, S. (2015). Telephone emergency services of Europe: Survey on volunteer satisfaction and motivation; Ifotes. http://www.ifotes.org/what-wedo/projects/tesvolsat-survey-on-volunteer-satisfaction-and-motivation

[cit0038] Triandis, H. C. (2001). Individualism-collectivism and personality. *Journal of Personality*, 69(6), 907–924. 10.1111/1467-6494.696169 11767823

[cit0039] Trompetter, H. R., de Kleine, E., & Bohlmeijer, E. T. (2017). Why does positive mental health buffer against psychopathology? An exploratory study on self-compassion as a resilience mechanism and adaptive emotion regulation strategy. *Cognitive Therapy and Research*, 41(3), 459–468. 10.1007/s10608-016-9774-0 28515539 PMC5410199

[cit0040] Tugade, M. M., & Fredrickson, B. L. (2004). Resilient Individuals Use Positive Emotions to Bounce Back From Negative Emotional Experiences. *Journal of Personality and Social Psychology*, 86(2), 320–333. 10.1037/0022-3514.86.2.320 14769087 PMC3132556

[cit0041] Tugade, M. M., & Fredrickson, B. L. (2007). Regulation of positive emotions: Emotion regulation strategies that promote resilience. *Journal of Happiness Studies*, 8(3), 311–333. 10.1007/s10902-006-9015-4

[cit0042] Van ’T Foort, B., & Veldkamp, T. (2019). Jaarverslag 2018; De Luisterlijn. Retrieved from Amersfoort: https://www.deluisterlijn.nl/publicaties/jaarverslagen/284-jaarverslag-2018/file.html

[cit0043] Van ’T Foort, B., & Veldkamp, T. (2020). Jaarverslag 2019; De Luisterlijn. https://www.deluisterlijn.nl/publicaties/jaarverslagen/332-jaarverslag-2019/file.html

[cit0044] Willems, R. C. W. J., Drossaert, C. H. C., Vuijk, P., & Bohlmeijer, E. T. (2020). Impact of crisis line volunteering on mental wellbeing and the associated factors: A systematic review. *International Journal Of Environmental Research And Public Health*, 17(5), 1641. 10.3390/ijerph17051641 PMC7084397 32138360

[cit0045] Xanthopoulou, D., Bakker, A. B., Demerouti, E., & Schaufeli, W. B. (2012). A diary study on the happy worker: How job resources relate to positive emotions and personal resources. *European Journal of Work and Organizational Psychology*, 21(4), 489–517. 10.1080/1359432X.2011.584386

[cit0046] Yanay, G. V., & Yanay, N. (2008). The decline of motivation?: From commitment to dropping out of volunteering. *Nonprofit Management & Leadership*, 19(1), 65–77. 10.1002/nml.205

